# Alpha 1 Antitrypsin Regulates Trophoblast Syncytialization and Inflammatory Factor Expression

**DOI:** 10.3390/ijms23041955

**Published:** 2022-02-10

**Authors:** Kanoko Yoshida, Aruto Yano, Kazuya Kusama, Gen Ishikawa, Kazuhiro Tamura

**Affiliations:** 1 Department of Endocrine Pharmacology, Tokyo University of Pharmacy and Life Sciences, Tokyo 192-0392, Japan; y164241@toyaku.ac.jp (K.Y.); y171180@toyaku.ac.jp (A.Y.); 2 Department of Obstetrics, Miyagi Children’s Hospital, Sendai 989-3126, Japan; genishi.0508@gmail.com

**Keywords:** trophoblasts, syncytialization, alpha-1-antitrypsin (A1AT), FOS like 1, AP-1 transcription factor subunit (FOSL1), inflammatory factor

## Abstract

The serine protease inhibitor alpha1-antitrypsin (A1AT) may possess protective functions of impaired organs in a manner independent of its protease inhibitor activity. A1AT expression has been shown to fluctuate in patients with pregnancy-induced hypertension, which suggests that A1AT may play a role in the syncytialization of villous trophoblasts. A1AT expression was knocked down in primary trophoblasts. RNA was extracted from these cells and subjected to RNA-sequencing analysis to determine the levels of expression of markers of syncytialization and inflammation. In addition, A1AT protein was localized in trophoblastic cells in placental tissues. Knockdown of A1AT upregulated the expression of FOSL1 and markers of syncytialization, as well as cell fusion, whereas overexpression of A1AT had the opposite effects. FOSL1 overexpression stimulated syncytialization, similar to the effects of A1AT knock down. Inhibitors of p38MAPK and JNK reduce the expression of inflammatory factors, whereas a p38MAPK inhibitor suppressed FOSL1 expression. Collectively, these findings indicated A1AT may negatively regulate inflammatory responses by controlling the activation of p38MAPK and JNK, and that p38MAPK mediates trophoblast syncytialization by altering FOSL1 expression. Therefore, a dysfunction in A1AT could be responsible for abnormal placental formation and pregnancy-associated disorders.

## 1. Introduction

Alpha-1 antitrypsin (A1AT), a member of the serpin superfamily, is a serine protease inhibitor encoded by the *SERPINA1* gene. In patients with inflammatory diseases, this protein is synthesized primarily in hepatocytes as an acute-phase reaction and secreted into the sera [1]. Interestingly, a reduction in intracellular A1AT protein has been shown to exacerbate inflammatory responses at endometriosis-like lesions in mice [2] and to increase endoplasmic reticulum (ER) stress-induced adipokine production in cultured human adipocytes [3]. Furthermore, treatment with purified A1AT was found to inhibit inflammatory responses induced by proinflammatory cytokines in human endometrial cells in vitro [2], and to improve the survival rate of mice with peritonitis and sepsis in vivo [4]. Although A1AT has been reported to reduce organ damage independent of its serine protease activity [5,6,7,8], the roles of A1AT in inflammatory physiology and disorders remain incompletely understood.

The placenta is the essential organ for the maintenance of pregnancy, with functions that include the exchange of gases and nutrition, the production of various bioactive factors and the maintenance of immune tolerance of the conceptus to the maternal immune system [9,10]. The placenta is composed primarily of fetal chorion and endometrial decidua. Human trophoblasts differentiate into villous and extravillous trophoblasts (EVT) [11]. During the process of syncytialization, cytotrophoblasts fuse to form multinucleate syncytiotrophoblasts, which are characterized by morphological and functional differentiation. In both primary human trophoblasts and the trophoblast-derived choriocarcinoma cell line BeWo, intracellular cyclic AMP (cAMP) mediates the production of human chorionic gonadotropin (hCG) and progesterone [12,13], and upregulates fusogenic syncytins through the transcription factors, glial cell missing 1 (GCM1), and ovo like transcriptional repressor 1 (OVOL1) [14,15,16].

Pathological conditions such as preeclampsia may be caused by insufficient invasion and syncytialization of trophoblasts [17,18]. A1AT has been shown to regulate ER stress-induced invasion of EVT [7], and urinary A1AT may be a marker of the severity of preeclampsia [19]. A1AT expression has been shown to fluctuate in patients with pregnancy-induced hypertension, which suggests that A1AT may play a role in the syncytialization of villous trophoblasts [20]. However, the roles of A1AT in placental physiology and disorders remain unknown. We therefore hypothesized that A1AT may play a role in the placental inflammatory response and trophoblast syncytialization. To test this hypothesis, we explored the role of A1AT on the expression of inflammatory cytokines and syncytialization markers in human trophoblast cells using RNA-sequencing (RNA-seq) analysis.

## 2. Results

### 2.1. A1AT Knockdown Upregulates Inflammatory Response-Related Gene Expression in Trophoblasts

**Figure 1:**
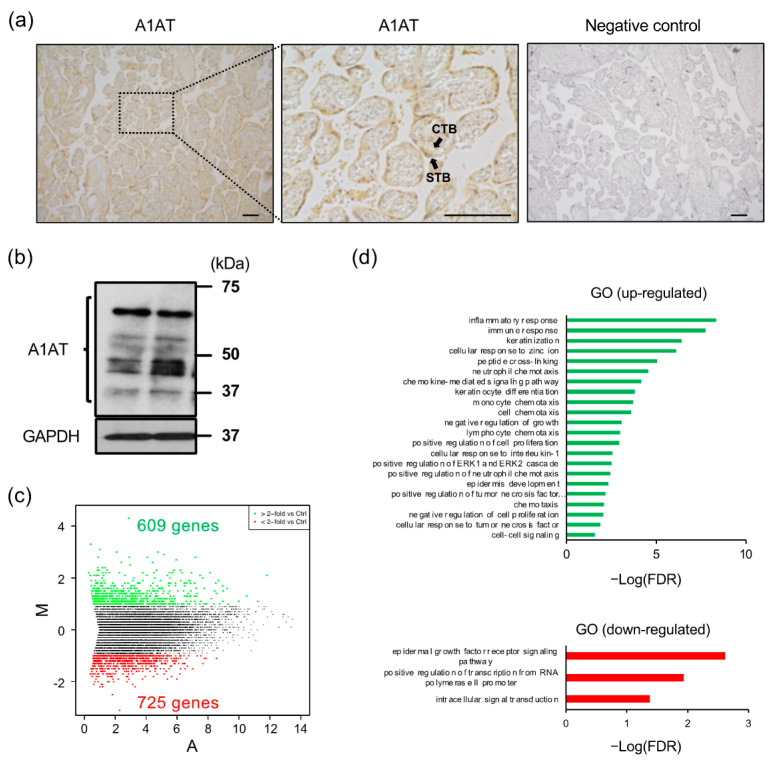
Knockdown of A1AT upregulates the expression of inflammatory response-related genes in trophoblasts. (**a**) Expression of A1AT protein in human placentas. Immunostaining was performed using anti-A1AT antibody or normal rabbit IgG (negative control). The pictures display the chorionic villi. The magnified middle picture shows the rectangle in the left panel. Scale bar = 100 μm. CTB: cytotrophoblasts, STB: syncytiotrophoblast. (**b**) Lysates prepared from primary trophoblasts were subjected to immunoblotting, with GAPDH serving as the loading control. (**c**) MA plot showing the expression of transcripts identified by RNA-seq. The transcripts highlighted in red or green were more than 2-fold differentially expressed (*p* < 0.05). (**d**) Functional classification of differentially expressed genes (DEGs) by Gene Ontology analysis.

Immunostaining of placental tissues with antibody to A1AT showed that A1AT was localized to cytotrophoblasts and syncytiotrophoblasts (Figure 1a), and western blotting showed that isolated trophoblasts express several molecular weights of A1AT, presumably glycosylated and cleaved forms (Figure 1b). The function of A1AT in trophoblasts was investigated by A1AT knockdown, followed by RNA-seq analysis of these trophoblast cells. A total of 1334 differentially expressed genes (DEGs) were detected (Figure 1c), with gene ontology (GO) biological process analysis showing that many of the upregulated DEGs were associated with the inflammatory response (Figure 1d).

### 2.2. A1AT Regulates Activation of p38MAPK and JNK and Expression of Inflammatory Cytokines in Trophoblasts

**Figure 2:**
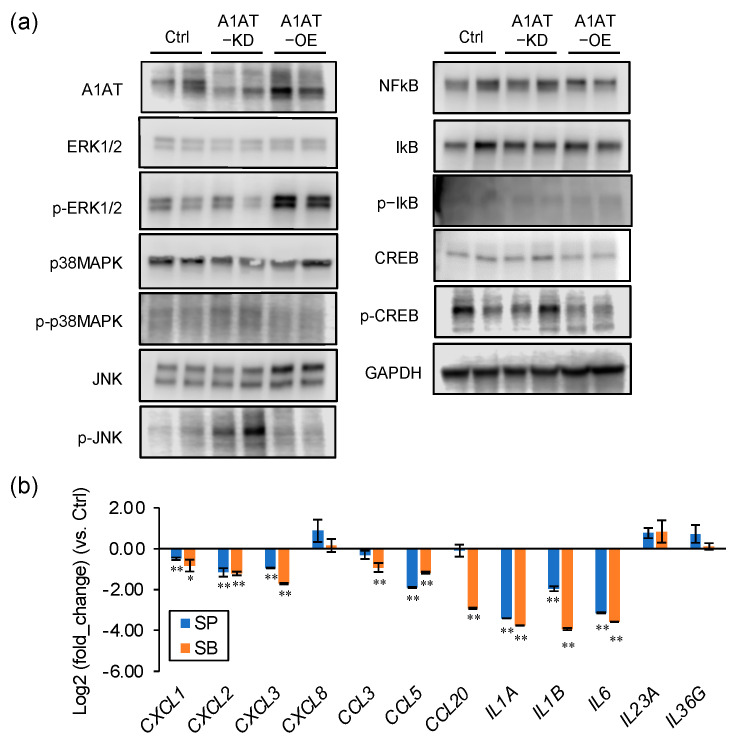
A1AT expression alters the levels of p38MAPK and JNK and the expression of inflammatory cytokines in trophoblasts. (**a**) Lysates prepared from BeWo cells with A1AT knockdown (A1AT-KD) or A1AT overexpression (A1AT-OE) were subjected to immunoblotting. GAPDH served as a loading control. (**b**) A1AT-KD BeWo cells were treated with the JNK inhibitor SP600125 (SP, 20 μM) or the p38 MAPK inhibitor SB203580 (SB, 20 μM) for 24 h, and the levels of mRNA encoding inflammatory cytokines were measured and normalized to that of *GAPDH* mRNA. The results shown are the means ± SEMs of three independent experiments. * *p* < 0.05, ** *p* < 0.01.

To investigate the mechanism by which A1AT regulates inflammatory responses in trophoblasts, A1AT was knocked down or overexpressed in the trophoblast BeWo cell line, and the levels of several intracellular signaling factors determined. The levels of phosphorylated JNK and p38MAPK were increased by A1AT knockdown and reduced by A1AT overexpression (Figure 2a). A1AT overexpression enhanced ERK1/2 phosphorylation, but neither A1AT knockdown nor overexpression altered the levels of phosphorylated NFKB, IKB, and CREB. To determine whether JNK and p38MAPK mediated the A1AT-induced expression of inflammatory cytokines, BeWo cells with knocked down A1AT were treated with inhibitors of JNK (SP600125) and p38MAPK (SB203580). Each inhibitor suppressed the expression of *CXCL1, CXCL2, CXCL3, CCL5, IL1A, IL1B*, and *IL6* mRNAs, all of which were inflammatory response-related DEGs (Figure 2b). The expression of *CCL3* and *CCL20* was inhibited by the p38MAPK inhibitor SB203580, but not by the JNK inhibitor SP600125.

### 2.3. A1AT Expression Alters the Syncytialization of Trophoblasts

**Figure 3:**
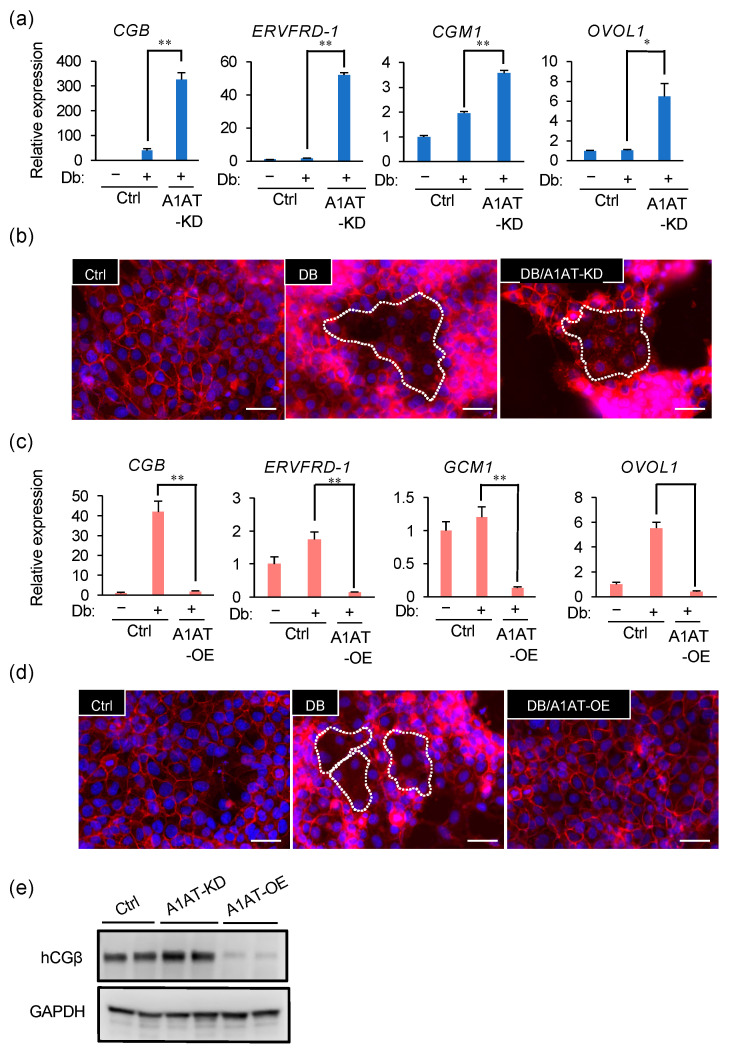
A1AT expression alters the syncytialization of trophoblasts. (**a**) A1AT-KD BeWo cells were treated for 72 h with Db (0.5 μM). Changes in syncytialization markers CGB, *ERVFRD-1*, *GCM1*, and *OVOL1* mRNA expression were determined by qPCR. The results shown are the means ± SEMs of three independent experiments. * *p* < 0.05, ** *p* < 0.01. (**b**) A1AT-KD BeWo cells were treated for 72 h with Db (0.5 μM). Cells were immunostained with anti-E-cadherin antibody (red) and DAPI (blue) to visualize syncytialization. A representative picture from three independent experiments is shown and the syncytialized cells are marked with a stippled line. Scale bar = 50 μm. (**c**,**d**) A1AT-OE BeWo cells were treated for 72 h with Db (0.5 μM), and the expression of mRNAs encoding the syncytialization markers described in (**a**) was determined by qPCR (**c**). The results are the means ± SEMs of three independent experiments. * *p* < 0.05, ** *p* < 0.01. Cells were immunostained to visualize syncytialization (**d**) as in (**b**). A representative picture from three independent experiments is shown. Scale bar = 50 μm. (**e**) Immunoblottingshowing the protein levels of the syncytialization marker hCGβ in lysates from A1AT-KD and A1AT-OE BeWo cells. GAPDH served as the loading control.

We next investigated whether A1AT has a significant effect on the syncytialization of trophoblasts. In the presence of a stable cAMP analog dibutyryl cAMP (Db), an inducer of syncytialization, A1AT knockdown further increased the expression of the syncytialization markers *CGB*, *ERVFRD-1*, *GCM1*, and *OVOL1* (Figure 3a) and enhanced cell fusion in BeWo cells (Figure 3b). Conversely, A1AT overexpression reduced the expression of Db-induced syncytialization markers and cell fusion (Figure 3c,d). The protein concentration of hCGβ, which is encoded by *CGB*, was also increased by A1AT knockdown and reduced by A1AT overexpression (Figure 3e).

### 2.4. FOSL1 Mediates A1AT Knockdown-Induced Syncytialization of Trophoblasts

**Figure 4:**
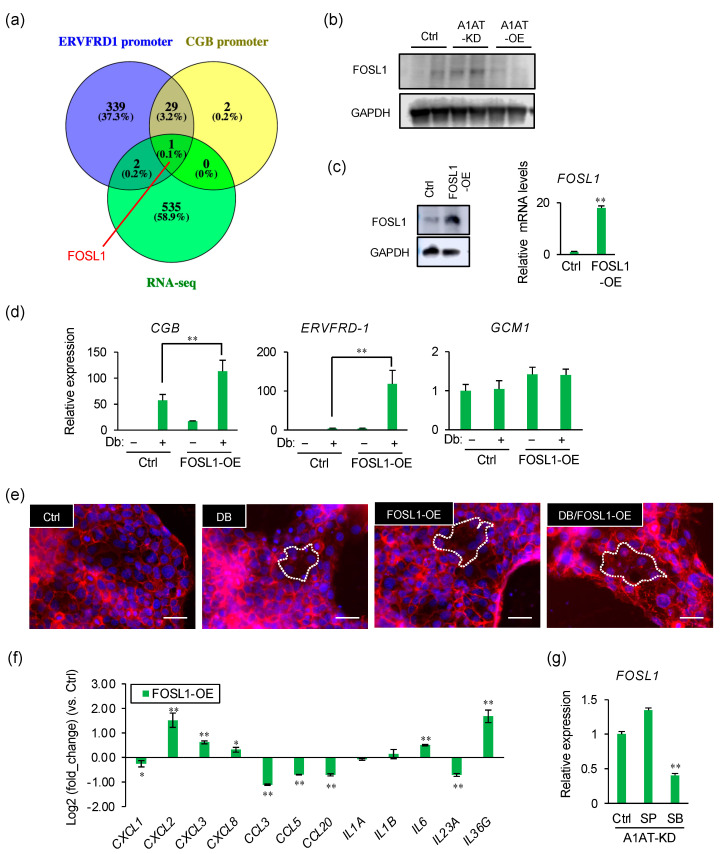
FOSL1 mediates A1AT knockdown-induced syncytialization of trophoblasts. (**a**) Venn diagram showing the numbers of upregulated DEGs and of genes encoding proteins that may be capable of binding to the *ERVFDR-1* or *CGB* promoter. (**b**) Immunoblotting showing the protein levels of FOSL1 in lysates from A1AT-KD and A1AT-OE BeWo cells. GAPDH served as the loading control. (**c**) Expression of FOSL1 in FOSL1-OE BeWo cells by Immunoblotting (left) and qPCR (right). Results shown are the means ± SEMs of three independent experiments. ** *p* < 0.01. (**d**) Expression of syncytialization markers in FOSL1-OE BeWo cells treated with Db (0.5 μM) by qPCR. Values are means ± SEMs of three independent experiments. ** *p* < 0.01. (**e**) Visualization of syncytialization by immunostaining cells with anti-E-cadherin antibody (red) and DAPI (blue). Representative pictures are shown, with syncytialized cells marked with a stippled line. Scale bar = 50 μm. (**f**) Expression of mRNAs encoding inflammatory cytokines in FOSL1-OE BeWo cells. *GAPDH* was used as the loading control. Results are reported as the means ± SEMs of three independent experiments. * *p* < 0.05, ** *p* < 0.01. (**g**) Expression of mRNAs encoding inflammatory cytokines in A1AT-KD BeWo cells treated with SP600125 (SP, 20 μM) or SB203580 (SB, 20 μM) for 24 h. *GAPDH* was used as the loading control. Results are reported as the means ± SEMs of three independent experiments. ** *p* < 0.01.

To identify the transcription factors that regulate the expression of syncytialization markers, we compared the genes previously reported to regulate ERVFRD-1 or CGB expression with the DEGs upregulated in our RNA-seq data. Only one gene was identified, *FOSL1* (Figure 4a). The level of FOSL1 protein was increased by A1AT knockdown and reduced by A1AT overexpression (Figure 4b). To evaluate its effect on syncytialization, FOSL1 was overexpressed in BeWo cells (Figure 4c). FOSL1 overexpression increased the Db-induced expression of *CGB* and *ERVFRD-1* (Figure 4d), and enhanced cell fusion (Figure 4e). In addition, FOSL1 overexpression increased the levels of expression of several inflammatory cytokines, such as *CXCL2*, *CXCL3*, *CXCL8*, *IL6*, and *IL36G* (Figure 4f). The effect of A1AT knockdown on the expression of *FOSL1* was blocked by the inhibition of p38MAPK (Figure 4g).

## 3. Discussion

**Figure 5:**
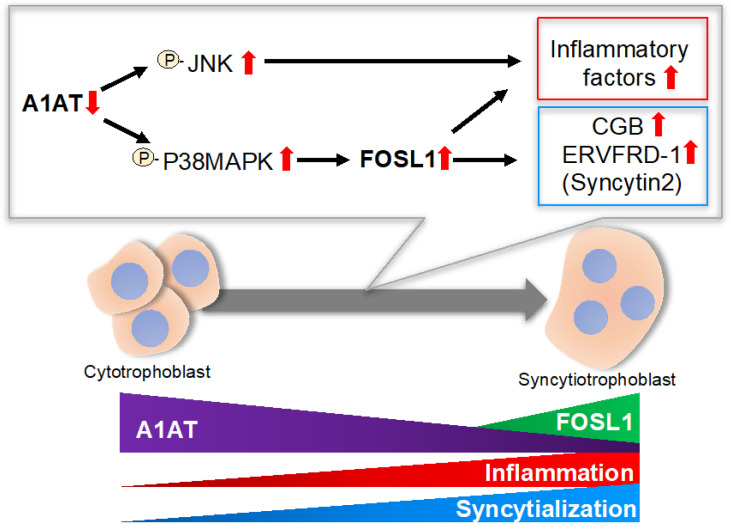
Schematic diagram illustrating the proposed A1AT/FOSL1 signaling in trophoblasts. A1AT controls the phosphorylation of p38MAPK and JNK, which may result in upregulation of several inflammatory cytokines, whereas p38MAPK regulates syncytialization by inducing FOSL1. P: phosphorylation. Red arrow: expression levels.

The present study showed that endogenous A1AT is associated with syncytialization via FOSL1, and that A1AT suppresses the expression of inflammatory cytokines in trophoblasts. A1AT knockdown enhanced, whereas A1AT overexpression inhibited, syncytialization. This study also found that the silencing of A1AT induced the expression of a large number of DEGs in primary trophoblasts, with most of these DEGs being immune-related genes. Knockdown of A1AT was found to stimulate the intracellular signaling proteins, p38MAPK and JNK, with their inhibitors abrogating the expression of inflammatory cytokines induced by A1AT silencing. Thus, a reduction in endogenous A1AT may lead to upregulation of the expression of FOSL1, which can promote the syncytialization of trophoblasts or the expression of several inflammatory cytokines via activation of p38MAPK (Figure 5).

A1AT is a serine protease inhibitor that might interact with the enzyme high-temperature requirement A serine peptidase 1 (HTRA1) [21]. However, preliminary results from our laboratory found that HTRA1 was not expressed in BeWo cells. A1AT expression in EVT has been reported to alter cell motility by inducing ER stress and HTRA1 in a serine protease activity-independent manner [7]. Neither A1AT knockdown nor overexpression altered the expression of ER stress markers in BeWo cells (data not shown). Importantly, the present study showed that A1AT knockdown activated p38MAPK and JNK. A1AT administration has been reported to inactivate the p21-activated kinase (PAK)/signal transducer and activator of transcription 1 (STAT1)/p38MAPK signaling pathway in trophoblasts from patients with preeclampsia [22,23], and S-nitrosylated A1AT was found to enhance the expression of inflammatory factors by activating p38MAPK and JNK [24]. In this study, several forms of A1AT protein were detected in villous lysates. Because A1AT activity may be influenced by post-translational modification, including S-nitrosylation, oxidization, glycosylation, and enzyme cleavage, the profile of A1AT forms might change under pathological placental conditions and consequently result in the functional alteration of this protein. In addition, it is reported that several protein kinases including MAPK play a crucial role in trophoblast differentiation and onset of preeclampsia [25,26,27]. These findings are consistent with our results, showing that A1AT regulates the syncytialization and expression of inflammatory cytokines via activation of p38MAPK and JNK.

FOSL1, a leucine zipper transcription factor and member of the activator protein 1 (AP-1) family, has been found to heterodimerize with JUN proteins. FOSL1 is thought to regulate fundamental cellular processes, including cell proliferation, differentiation, motility, and invasion [28]. *Fosl1* knockout in rats resulted in early embryonic death [29]. Evaluation of rat trophoblast stem cells showed that FOSL1 expression was markedly increased during trophoblast differentiation, with these cells acquiring both endocrine and invasive properties [20,29,30,31]. The role of FOSL1 in trophoblast invasion is conserved in rats and human trophoblast cells [29,31,32].

This study found that knockdown of A1AT upregulated the expression of FOSL1. Similar to A1AT knockdown, FOSL1 overexpression also increased the expression of syncytialization markers and cell fusion. Furthermore, p38MAPK inhibitor downregulated FOSL1 expression induced by A1AT knockdown. Taken together, these findings indicate that a decrease in A1AT upregulated FOSL1 expression via the activation of p38MAPK, resulting in the syncytialization of trophoblasts. FOSL1 overexpression also induced the expression of several proinflammatory cytokines, suggesting that the expression of inflammatory cytokines induced by knocking down A1AT was partially mediated by FOSL1.

In conclusion, this study demonstrated that A1AT regulated the expression in trophoblasts of inflammatory cytokines via the activation of p38MAPK and JNK, and that A1AT enhanced the syncytialization of trophoblasts via the expression of FOSL1 induced by p38MAPK. These findings suggest that optimal expression of A1AT in trophoblasts may be required for placental syncytialization and inflammatory responses, and that villous A1AT expression may be associated with hypertensive disorders of pregnancy and early abortion.

## 4. Materials and Methods

### 4.1. Immunohistochemistry

Placental tissues were obtained from three pregnant women during their third trimester (32 weeks of gestation) in Nippon Medical School. Informed consent was obtained from each patient who underwent cesarean section, and the study protocol was approved by the institutional review board of Nippon Medical School and the Clinical Research Ethics Committee of Tokyo University of Pharmacy and Life Sciences (#1512: approval 1 September 2015). The sections were immunostained for A1AT. Briefly, portions of the placental samples were immediately fixed in 4% paraformaldehyde in PBS, dehydrated, and embedded in paraffin. The sections were rehydrated, boiled for 20 min in 10 mM citrate buffer (pH 6.0), and incubated overnight at 4 °C with rabbit polyclonal anti-A1AT antibody (1:200; Dako, Tokyo, Japan) or, as a negative control, an equal molar concentration of normal rabbit IgG (1:100, sc-2027; Santa Cruz Biotechnology, Dallas, TX, USA), which were incubated with Histofine Simple Stain MAX-PO (Nichirei Biosciences, Tokyo, Japan). The sections were stained with DAB (Fujifilm Wako Pure Chemical Corp., Osaka, Japan), and counterstained with hematoxylin [7].

### 4.2. Cell Culture

After thoroughly washing the placental samples with Ca^2+^/Mg^2+^-free Hank’s solution to remove blood, the samples were minced with scissors and incubated for 20 min with 0.25% trypsin and 0.25% collagenase Type IA (Sigma-Aldrich, St. Louis, MO, USA) in a 37 °C water bath with continuous gentle shaking. The dispersed cells were strained through a 250 μm sterilized sieve (Sanpo, Tokyo, Japan) to remove undigested and mucosal tissues. Highly purified trophoblasts were obtained using the Percoll gradient method, as described [33], seeded in wells of a 24-well tissue culture plate coated with 0.1% Cellmatrix^®^ Type IA (Nitta Gelatin Inc., Osaka, Japan) in a 1:1 mixture of Ham’s F12 and Dulbecco’s modified Eagle’s medium (Fujifilm Wako Pure Chemical Corp.) supplemented with 20% (*v*/*v*) fetal bovine serum (FBS; Nichirei Biosciences), 100 ng/mL epidermal growth factor (PeproTech Inc., Rocky Hill, NJ, USA), 1% PSN (100 μg/mL penicillin, 100 μg/mL streptomycin, and 200 μg/mL neomycin; Thermo Fisher Scientific, Waltham, MA, USA), and 0.5 μg/mL amphotericin B; and maintained overnight at 37 °C in humidified air containing 5% CO_2_.

The human choriocarcinoma BeWo cell line, purchased from the JCRB Cell Bank (Osaka, Japan), were grown in 1:1 Ham’s F12/Dulbecco’s modified Eagle’s medium (Fujifilm Wako Pure Chemical Corp.) supplemented with 10% FBS and 1% PSN at 37 °C in humidified air containing 5% CO_2_ [34]. Where indicated, the cells were treated with dibutyryl cAMP (Db, 500 μM, Tokyo Chemical Industry, Tokyo, Japan) for 72 h to induce cell differentiation. These cells were also treated for 24 h with the p38 MAPK inhibitor SB203580 (20 μM, Selleck Chemicals, Tokyo, Japan) or the JNK inhibitor SP600125 (20 μM, Selleck Chemicals).

### 4.3. Western Blotting

Harvested cells were lysed in RIPA buffer (Thermo Fisher Scientific), and equal amounts of lysate proteins were separated by SDS-PAGE and transferred to polyvinylidene difluoride membranes (Bio-Rad Laboratories, Hercules, CA, USA) using a Trans-Blot Turbo (Bio-Rad). After blocking with Bullet Blocking One (Nacalai Tesque, Kyoto, Japan), the membranes were incubated with primary antibodies against A1AT (1:2000; Dako, Tokyo, Japan), ERK1/2 (1:2000, #4695; Cell Signaling Technology [CST], Tokyo, Japan), phosphorylated (p-) ERK1/2 (1:2000, #4370; CST), p-38MAPK (1:2000, #8690; CST), p-p38MAPK (1:2000, #4511; CST), JNK (1:2000, #9252; CST), p-JNK (1:2000, #9251; CST), NFkB (1:2000, #4764; CST), IkB (1:2000, #4814; CST), p-IkB (1:2000, #2859; CST), CREB (1:2000, #9197; CST), p-CREB (1:2000, #9198; CST) hCGB (Thermo Fisher Scientific), or GAPDH (1:5000, 5A12; Fujifilm Wako Pure Chemical Corp.). After washing, the membranes were incubated with horseradish peroxidase-linked goat anti-rabbit or anti-mouse IgG (1:5000; Vector Laboratories, Burlingame, CA, USA). Immunoreactivity was detected with a chemiluminescence (Merck Millipore, Burlington, MA, USA) [7].

### 4.4. Transfection of Small Interfering (si)RNA

BeWo cells were transfected with either non-targeting control or *SERPINA1* siRNA (50 nM, EHU090971, Sigma-Aldrich) using Lipofectamine RNAiMAX (Thermo Fisher Scientific), according to the manufacturer’s instructions [7].

### 4.5. RNA-Sequencing (RNA-Seq), GO, and Pathway Analyses

For RNA-seq analysis, RNA was extracted from cultured primary trophoblast cells using Isogen II (Nippon Gene), according to the manufacturer’s instructions. The cDNA libraries for RNA-seq were prepared using a TruSeq Stranded mRNA LT Sample Prep Kit (Illumina, San Diego, CA, USA), according to the manufacturer’s instructions, and data were analyzed by Macrogen Japan (Kyoto, Japan). Output data were deposited in the DDBJ (DNA Data Bank of Japan) Sequence Read Archive (https://www.ddbj.nig.ac.jp/dra/index-e.html (accessed on 1 November 2021) under accession numbers DRR304274 and DRR304275). Data were analyzed as described [35]. Briefly, trimmed sequences were analyzed using the TopHat/Cufflinks pipeline, the human genome (hg38), and reference annotations obtained from the UCSC genome browser (https://genome.ucsc.edu (accessed on 1 November 2021)). DEGs were categorized by gene-level FPKM (fragments per kilobase of exon per million mapped fragments) expression levels. Genes with an absolute expression level of >2 FPKM were selected. GO analyses were performed using the Enrichr tool (http://amp.pharm.mssm.edu/Enrichr/ (accessed on 1 November 2021)).

### 4.6. RNA Extraction and Quantitative RT-PCR

RNA was extracted from cells using RNeasy Mini Kits (Qiagen, Tokyo, Japan), according to the manufacturer’s instructions, and the resulting mRNA was reverse transcribed using a ReverTra Ace qPCR RT Kit (Toyobo, Osaka, Japan). Sequences in the resulting cDNA were subjected to qPCR amplification using specific primers (Table 1) and a PowerUP SYBR Green Master Mix (Thermo Fisher Scientific). Calibration curves were used to determine the level of expression of each target gene relative to that of a reference gene, glyceraldehyde-3-phosphate dehydrogenase (*GAPDH*). The mean crossing threshold (Ct) for each target was calculated using Sequence Detection System software v2.3 (Thermo Fisher Scientific) [35].

### 4.7. Transfection of the Expression Plasmid Construct

The SERPINA1 (A1AT) expression vector pTCP (BC011991) was purchased from TransOMIC Technologies (Huntsville, AL, USA). The FOSL1 expression vector pRP (pDNA VB900007-4466mvj) was purchased from VectorBuilder (Chicago, IL, USA). The pTCP-SERPINA1 or pRP-FOSL1 plasmid (1 μg) was transfected into BeWo cells by electroporation using the Neon transfection system (Thermo Fisher Scientific), according to the manufacturer’s instructions. Cells were pulsed twice with 1400 V for 20 ms, and transfected cells were selected using puromycin (3 μg/mL) for 48 h [7].

### 4.8. Cell Fusion Assay

BeWo cells were fixed with methanol and incubated with anti-E-cadherin antibody (1:200, #3195, CST) and AlexaFluor 594-conjugated goat anti-mouse antibody (Thermo Fisher Scientific) to distinguish cell surfaces. The nuclei were counterstained with 4′,6-diamino-2-phenylindole 2HCl (DAPI). The numbers of multinucleated cells in five randomly selected microscopic areas per sample were counted and evaluated in three independent experiments [34].

### 4.9. Statistical Analysis

Data are expressed as mean ± SEM and compared using Dunnett’s test. All statistical analyses were performed using R software (ver.4.0.5; www.r-project.org, accessed on 1 November 2021), with *p*-values < 0.05 considered statistically significant.

## Figures and Tables

**Table 1:** Primers for real-time qPCR analyses.

Name (Accession No.)	Sequence (5′---3′)	Product Length (bp)
*GAPDH* NM_002046.7	AGCCACATCGCTCAGACA	66
	GCCCAATACGACCAAATCC	
*CXCL1* NM_001511.4	AGCTTGCCTCAATCCTGCATCC	119
	TCCTTCAGGAACAGCCACCAGT	
*CXCL2* NM_002089.4	GGCAGAAAGCTTGTCTCAACCC	127
	CTCCTTCAGGAACAGCCACCAA	
*CXCL3* NM_002090.3	TTCACCTCAAGAACATCCAAAGTG	94
	TTCTTCCCATTCTTGAGTGTGGC	
*CXCL8*	GAGAGTGATTGAGAGTGGACCAC	112
NM_000584.4	CACAACCCTCTGCACCCAGTTT	
*CCL3*	ACTTTGAGACGAGCAGCCAGTG	101
NM_002983.3	TTTCTGGACCCACTCCTCACTG	
*CCL5*	CCTGCTGCTTTGCCTACATTGC	125
NM_002985.3	ACACACTTGGCGGTTCTTTCGG	
*CCL20*	AAGTTGTCTGTGTGCGCAAATCC	107
NM_004591.3	CCATTCCAGAAAAGCCACAGTTTT	
*IL1A*	TGTATGTGACTGCCCAAGATGAAG	96
NM_000575.5	AGAGGAGGTTGGTCTCACTACC	
IL1B	TGATGGCTTATTACAGTGGCAATG	140
NM_000576.3	GTAGTGGTGGTGGGAGATTCG	
*IL6*	CAGGAGCCCAGCTATGAACT	85
NM_000600.5	AGCAGGCAACACCAGGAG	
*IL23A*	GAGCCTTCTCTGCTCCCTGATA	121
NM_016584.3	GACTGAGGCTTGGAATCTGCTG	
*IL36G*	GAAGGTTGGAGAACAGCCCACA	130
NM_019618.4	AGACTCAAGGGTGGAGGTCCTA	
*CGB*	CCTGGCCTTGTCTACCTCTT	108
NM_000737.3	GGCTTTATACCTCGGGGTTG	
*ERVFRD-1*	CCAAATTCCCTCCTCTCCTC	115
NM_207582.2	CGGGTGTTAGTTTGCTTGGT	
GCM1	GCAACACCAACAACCACAAC	100
NM_003643.3	GTAAATCTTGCGGCCTTCCT	
OVOL1	AGACATGGGCCACTTGACAG	104
NM_004561.3	AGGTGAACAGGTCTCCACTG	
FOSL1	GGAGGAAGGAACTGACCGACTT	113
NM_005438.5	CTCTAGGCGCTCCTTCTGCTTC	

## Data Availability

RNA-seq data were deposited in the DDBJ (DNA Data Bank of Japan) Sequence Read Archive (https://www.ddbj.nig.ac.jp/dra/index-e.html, accessed on 14 December 2021) under accession numbers DRR304274 and DRR304275).
